# Osteoclast Signal Transduction During Bone Metastasis Formation

**DOI:** 10.3389/fcell.2020.00507

**Published:** 2020-06-19

**Authors:** Dávid S. Győri, Attila Mócsai

**Affiliations:** Department of Physiology, Faculty of Medicine, Semmelweis University, Budapest, Hungary

**Keywords:** osteoclast (OC), signaling/signaling pathways, tumor, bone metastases (BM), osteolysis

## Abstract

Osteoclasts are myeloid lineage-derived bone-resorbing cells of hematopoietic origin. They differentiate from myeloid precursors through a complex regulation process where the differentiation of preosteoclasts is followed by intercellular fusion to generate large multinucleated cells. Under physiological conditions, osteoclastogenesis is primarily directed by interactions between CSF-1R and macrophage colony-stimulating factor (M-CSF, CSF-1), receptor activator of nuclear factor NF-κB (RANK) and RANK ligand (RANKL), as well as adhesion receptors (e.g., integrins) and their ligands. Osteoclasts play a central role in physiological and pathological bone resorption and are also required for excessive bone loss during osteoporosis, inflammatory bone and joint diseases (such as rheumatoid arthritis) and cancer cell-induced osteolysis. Due to the major role of osteoclasts in these diseases the better understanding of their intracellular signaling pathways can lead to the identification of potential novel therapeutic targets. Non-receptor tyrosine kinases and lipid kinases play major roles in osteoclasts and small-molecule kinase inhibitors are emerging new therapeutics in diseases with pathological bone loss. During the last few years, we and others have shown that certain lipid (such as phosphoinositide 3-kinases PI3Kβ and PI3Kδ) and tyrosine (Src−family and Syk) kinases play a critical role in osteoclast differentiation and function in humans and mice. Some of these signaling pathways shows similarity to immunoreceptor-like receptor signaling and involves important other enzymes (e.g., PLCγ2) and adapter proteins (such as the ITAM−bearing adapters DAP12 and the Fc-receptor γ-chain). Here, we review recently identified osteoclast signaling pathways and their role in osteoclast differentiation and function as well as pathological bone loss associated with osteolytic tumors of the bone. A better understanding of osteoclast signaling may facilitate the design of novel and more efficient therapies for pathological bone resorption and osteolytic skeletal metastasis formation.

## Development and Function of Osteoclasts and Their Role in Pathological Bone Loss

Bone tissue plays a crucial role in structural support and movement of the body as well as it stores minerals. It also hosts the bone marrow, which is the major site of postnatal hematopoiesis (Zaidi, 2007). Bone matrix is an essential component of the bone and it is built up from inorganic salts and organic matrix. Besides providing structural support, bone matrix also stores a wide range of growth factors capable of regulating normal bone homeostasis. Bone microenvironment contains a wide repertoire of cellular elements: hematopoietic and mesenchymal stem cells, chondrocytes, fibroblasts, adipocytes, endothelial and nerve cells as well as the bone cells themselves (Arron and Choi, 2000). The most well-known of these latter ones are bone-resorbing osteoclasts, bone-forming osteoblasts and osteocytes regulating the bone remodeling process (Bonewald, 2011).

Osteoclasts are derived from myeloid precursors, which express several cytokine receptors. Osteoclast differentiation is mainly governed by receptor activator of NF-κB ligand (RANKL) and macrophage colony-stimulating factor (M-CSF or CSF-1), as well as integrin and immunoreceptor-like adhesion signals and interactions, which are provided by osteoclastogenesis-supporting cells, such as osteoblasts, osteocytes and other stromal cells under physiological conditions (Boyle et al., 2003). The early phase of osteoclast differentiation is characterized by the expression of osteoclast-specific genes, such as tartrate-resistant acidic phosphatase (TRAP) in committed precursors (preosteoclasts). Fusion of these preosteoclasts will then lead to formation of large, multinucleated osteoclasts. These giant polykarions spread over the bone surface and digest the underlying bone tissue through the simultaneous release of hydrochloric acid and digestive enzymes onto the bone (Teitelbaum, 2000).

The tightly regulated balance between bone resorption and bone formation can be altered under pathological conditions. Enhanced maturation and activation of osteoclasts leads to pathological bone resorption as seen in osteoporosis and inflammatory bone diseases (Győri and Mócsai, 2015). During the pathogenesis of inflammatory bone diseases such as rheumatoid arthritis, gout and periodontitis, the chronic inflammation irreversibly affects the surrounding bone tissue. Bone degradation in inflammatory arthritis is best characterized in human rheumatoid arthritis (Győri and Mócsai, 2015). Rheumatoid arthritis is a chronic autoimmune disease, eventually leading to the destruction of surface cartilage and subchondral bone primarily in the small synovial joints of the hands and feet (Firestein, 2003). Osteoclasts play a key role in the pathogenesis of rheumatoid arthritis (McInnes and Schett, 2007) and numerous studies focused on the crosstalk between osteoclast and the immune system in rheumatoid arthritis. Mature osteoclasts are present at the sites of bone destruction and osteoclastogenesis is enhanced in the close proximity of the inflamed joints (Schett and Teitelbaum, 2009). This pronounced osteoclast formation is due to the accumulation of osteoclast precursors at the sites of erosion and enhanced maturation of these preosteoclasts to bone-resorbing polykarions in the presence of osteoclastogenic cytokines derived from the immune and stromal cells (Schett, 2009). Besides inflammatory bone diseases and osteoporosis, the third major disease where excessive bone loss occurs due to hyperactivation of osteoclasts is tumor-induced osteolysis and the formation of bone metastases.

## Role of Osteoclasts in Tumor-Induced Osteolysis and Bone Metastasis Formation

Bone tissue is one of the most common sites for metastasis formation by a large number of solid tumors including lung, prostate, breast, thyroid, colorectal, ovarian cancers, and malignant melanoma. Further, two-third of patients with stage II/III prostate and breast cancers develop bone metastasis (Hernandez et al., 2018). Bone metastases are classified as osteolytic, osteoblastic and mixed lesions. The role of osteoclasts and induction of osteoclastogenesis is best described in the process of osteolytic bone metastasis formation. The presence of osteolytic bone lesions are associated with a set of different morbidities including pathological fractures, pain and hypercalcemia (Weilbaecher et al., 2011), seriously affecting the patient’s wellbeing and life expectancy (Coleman and Rubens, 1987).

Although solid tumors capable of forming osteolytic lesions have proteolytic activity, the extent of this is far from being able to break down the bone matrix. Degradation of both the organic and inorganic components of the bone is therefore carried out by osteoclasts, the unique bone-resorbing cells, accumulating in the vicinity of tumor cells forming osteolytic metastases (Croucher et al., 2016). Tumor cells can promote osteoclast-mediated osteolysis via several mechanisms. Either tumor cells can induce osteoclast differentiation directly via the expression of RANKL or they can stimulate osteoclastogenesis indirectly via the activation of osteoblasts (Mundy, 2002). During this latter process, a wide range of tumor cell-derived growth factors such as parathyroid hormone related peptide – (PTHrP) can induce the expression of RANKL on osteoblasts, which in turn drives the differentiation of multinucleated osteoclasts from myeloid precursors (Suva et al., 1987). Mature osteoclasts then resorb the bone matrix and allow tumor cells to grow and spread within the tissue.

Skeletal metastases formation is a self-perpetuating cycle where tumor cells and bone-resorbing osteoclasts are enrolled in a “vicious” cycle characterized by the release of bone-stored growth factors by osteoclast-mediated bone resorption, which further stimulates cancer cell survival and proliferation (Faccio, 2011). Malignant cells express a wide range of growth factors and cytokines, which can directly or indirectly activate osteoclasts. On the other hand, osteoclast-mediated bone resorption can lead to the release of bone-stored cytokines including TGFβ, which are able to promote cancer cell survival and growth (Kakonen et al., 2002). Bone matrix-derived cytokines can also provide a chemotactic stimulus for directed cancer cell migration (Orr et al., 1979). Further, RANKL itself via a paracrine mechanism can serve as a chemoattractant and increase migration of RANK-positive cancer cells (Jones et al., 2006). As a consequence, osteoclast-mediated osteolysis results in an altered bone microenvironment, which facilitates cancer growth and metastasis formation. Later during the disease, these interactions between tumor and bone cells result in a locked cycle of tissue destruction and cancer growth (“vicious cycle” of bone metastasis formation) (Roodman and Dougall, 2008). In line with this, it has been found in mice, that cancer cells, which are more closely located to the bone surface showed increased proliferation compared to the ones distant from the bone (Kostenuik et al., 1992). Underlying the role of osteoclasts in the process of bone metastasis formation, bisphosphonate pyrophosphate analogs that target osteoclasts are used to prevent bone destruction and modify progression of skeletal metastasis in cancer (Choi et al., 2009).

Bone remodeling is also closely coupled with the lympho-hematopoietic system. The similarity between the signaling mechanism of the bone and immune systems in this shared microenvironment indicate that cancer cell growth associated with osteolytic bone degradation can also drive local immunosuppression and accumulation of metastasis-promoting immune cell populations (Lorenzo et al., 2007). A large body of experimental evidence has implicated the role of the immune system in the regulation of bone homeostasis both in humans and mice (Lorenzo et al., 2010).

## Interplay Between the Skeletal and Immune Systems During Bone Metastasis Formation

Tumor development can alter both the skeletal and immune homeostasis (Nakashima and Takayanagi, 2009). Malignant cells are able to suppresses certain effector immune cells subsets, such as conventional CD8^+^ T cells, which can recognize and kill cancer cells (Schreiber et al., 2011). Other immune cells, such as regulatory T cells (Tregs), myeloid-derived suppressor cells (MDSC) and tumor-associated macrophages (TAMs) also play important roles in promoting cancer growth and metastasis formation. On the other hand, the cellular elements and humoral factors of the innate and adaptive immune systems can affect osteoclastogenesis as well (Takayanagi, 2010). Macrophages and dendritic cells also share common precursors with osteoclasts, which underline the importance of the field of osteoimmunology (Takayanagi, 2007).

While Th1 and Th2 cytokines exert an inhibitory effect on osteoclastogenesis, the IL-17 producing T helper type 17 (Th17) cells have been described to be highly osteoclastogenic (Sato et al., 2006b). Th17 cells can express high levels of RANKL and as a consequence directly promote osteoclastogenesis. Moreover, they may activate inflammation locally, leading to the release of proinflammatory mediators (e.g., TNF-α, IL-1, and IL-6), which can potentiate RANKL expression on osteoclastogenesis-supporting cells (Sato et al., 2006b). Further, Th17 cells were described to activate osteoclastogenesis-driven osteolysis through RANKL production during inflammatory arthritis (Okamoto and Takayanagi, 2011). However, no direct role for Th17 cells in cancer induced bone disease has been reported so far. In line with this, IL-17F, which shows 50% homology with IL-17A and shares its receptor, was produced in high levels in the 4T1 preclinical tumor model, but found not to be necessary for the development of pre-metastatic bone disease (Monteiro et al., 2013).

The effects of conventional T cells on osteoclastogenesis are normally suppressed by regulatory T (Treg) cells. These cells are able to inhibit osteoclast development and function via the release of tumor growth factor-β (TGF-β), IL-10 (Kim et al., 2007b; Kelchtermans et al., 2009) and expression of CTLA-4 (Zaiss et al., 2007). In addition to their suppressive capabilities, tumor-infiltrating Treg cells have also been described to express RANKL (Tan et al., 2011). As a consequence, the effect of Treg cells on osteoclastogenesis depends on the balance between positive and negative factors within the tumor microenvironment.

Myeloid-derived suppressor cells (MDSCs) are a heterogeneous population of immature myeloid cells, which are capable of potently suppressing the anti-tumor functions of conventional T lymphocytes (Gabrilovich and Nagaraj, 2009). It has been shown that MDSCs derived from the tumor microenvironment can differentiate into bone-resorbing osteoclasts under tissue culture conditions (Sawant et al., 2013) and MDSCs from tumor-bearing mice have increased osteoclastogenic potential (Zhuang et al., 2012).

Tumor-associated macrophages (TAMs) are a predominant white blood cell subset both in the bone and tumor microenvironment, which can influence tumor development, proliferation, growth, survival, and metastasis formation. Macrophages classically have been divided into proinflammatory M1 and anti-inflammatory M2 subsets (Mosser and Edwards, 2008). When activated, M1 macrophages secrete high levels of proinflammatory cytokines and participate in the elimination of tumor cells (Gabrilovich et al., 2012). However, M2 macrophages are characterized by high expression of mannose receptors, scavenger receptors and IL-1Ra (Zhang et al., 2012), and are also often found in human solid tumors. Activated M2 macrophages generate high levels of IL-10 and TGF-β, which can suppress CD4^+^ and CD8^+^ conventional T cells (Biswas and Mantovani, 2010). In preclinical studies, where tumor-associated macrophages were depleted using clodronate liposomes, reduced number of bone metastatic lesions were detected (Hiraoka et al., 2008).

## Role of RANKL Signaling in Osteoclasts and Bone Metastasis Formation

Receptor activator of NF-κB ligand (RANKL) belongs to the tumor necrosis factor (TNF) superfamily of cytokines and it is expressed by monocytes, T and B cells, dendritic cells and osteoclastogenesis-supporting cells, such as osteoblasts and synovial fibroblasts (Caetano-Lopes et al., 2009). Parathyroid hormone, 1,25-dihydroxy-cholecalciferol (active vitamin D3) and prostaglandins can promote the secretion of RANKL by osteoblasts and other stromal cellular elements (Takayanagi, 2007). CD4^+^ conventional and regulatory T lymphocytes are also able to provide RANKL in membrane-bound form as well as release it in a soluble form (Wong et al., 1997). TNF-α, IL-1, IL-6 and IL-17 cytokines can increase RANKL expression on osteoclastogenesis-supporting cells, thereby stimulating RANKL signaling (Takayanagi, 2007). Osteoprotegerin (OPG), expressed by osteoblasts and other stromal cells, is a soluble decoy receptor for RANKL capable of inhibiting RANK signaling (Simonet et al., 1997).

**FIGURE 1:**
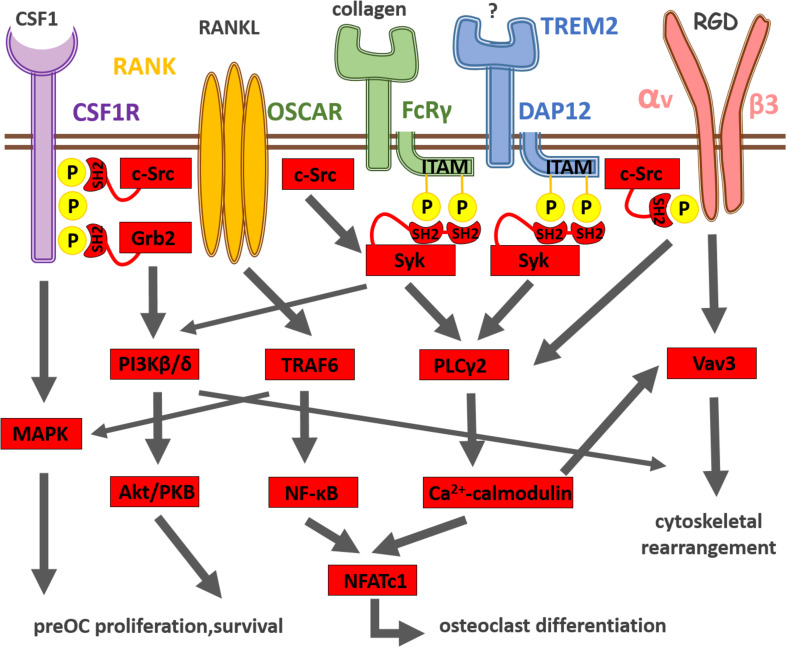
Summary of osteoclast differentiation induced by integration of CSF-1R, RANK, immunoreceptor-like and integrin receptor signaling. CSF-1 and its receptor CSF-1R activate the MAPK cascade pathway leading to the survival and proliferation of preosteoclasts. RANKL and its receptor RANK transduce signals via the adaptor molecule TRAF6, which activates the NF-κB and MAPK pathways leading to the differentiation of osteoclasts. The expression of the master regulator of osteoclastogenesis, NFATc1, is driven by NF-κB and NFATc1. The activation of NFATc1 is also regulated by the costimulatory signaling pathways, where FcRγ, DAP12 and their associating partners (OSCAR and TREM2, respectively) recruit Syk, which further activates PLCγ2, resulting in the activation of calcium signaling. The calcium signaling activates then calcineurin, which in turn promotes NFATc1 expression. The calcium signal also induces Vav3 activation involved in αvβ3 integrin signaling, which leads to cytoskeletal reorganization and osteoclastic bone resorption.

The RANKL receptor, RANK, is highly expressed by preosteoclast. RANKL binding to RANK leads to receptor trimerization and activation of the adapter protein TRAF6, which further stimulates transcription factor NF-κB and members of the mitogen-activated protein kinase (MAPK) family (Takayanagi, 2010) as shown on Figure 1. Nuclear factor of activated T-cell cytoplasmic 1 (NFATc1), the master regulator of osteoclast differentiation, is also activated by RANK receptor signaling (Takayanagi, 2010). NFATc1 translocates to the nucleus and then amplifies its own expression resulting in strong induction of NFATc1 expression (Asagiri et al., 2005). Generation of calcium signal and calcineurin activation are also important for NFATc1 induction. NFATc1 together with activator protein 1 (AP-1) and microphthalmia-associated transcription factor (MITF) induce then the expression of osteoclast-specific genes encoding for tartrate-resistant acid phosphatase (TRAP), cathepsin K (CTSK) and the β3 integrin (Takayanagi, 2007).

The expression of NFATc1 in osteoclasts and their precursors is also regulated on the epigenetic level. An important step in the differentiation of osteoclasts occurs at the NFATc1 promoter when the histone methylation changes from H3K4me3/H3K27me3 to H3K4me3 (Yasui et al., 2011). A histone demethylase, Jmjd3 converts the bivalent H3K4/H3K27 trimethylation to monovalent H3K4me3 in preosteoclasts following RANKL stimulation, leading to increased osteoclast differentiation as well (Yasui et al., 2011). On the other hand, NFATc1 expression can also be inhibited by other transcription factors such as Interferon regulatory factor 8 (IRF-8) (Zhao et al., 2009), bZIP motif containing transcription factor MafB (Kim et al., 2007a), B-cell lymphoma 6 (Bcl-6) (Miyauchi et al., 2010), and Leukemia/lymphoma related factor (LRF) (Tsuji-Takechi et al., 2012). The expression of those transcription factors decrease during osteoclast differentiation, which is mediated by the DNA methyl-transferase 3A (Dnmt3A) (Nishikawa et al., 2015). Furthermore, the B lymphocyte-induced maturation protein (Blimp) was shown to be able to inhibit IRF-8, MafB, Bcl-6 and LRZ transcription factors, leading to upregulated NFATc1 expression and enhanced osteoclastogenesis (Nishikawa et al., 2010). In line with this, Blimp1-deficient mice exhibit increased bone mass and osteopetrotic disease (Nishikawa et al., 2010).

Cancer cells enhance osteoclast-driven osteolysis via several different mechanisms. Upregulation of the expression of RANKL on osteoclastogenesis-supporting cells, downregulation of OPG expression or increased secretion of factors activating RANK receptor signaling have all been described in the context of breast cancer bone metastases (Kearns et al., 2008). Prostate tumor cells can even express RANKL themselves (Brown et al., 2001). Further, secretion of RANKL has also been described by multiple myeloma cells (Farrugia et al., 2003; Sezer et al., 2003). While breast tumors do not upregulate RANKL (Thomas et al., 1999), those cells can eventually induce the expression of RANKL on osteoblasts (Kitazawa and Kitazawa, 2002) and other osteoclastogenesis-supporting cells via the production and release of PTHrP (Mancino et al., 2001). RANK receptor expression by melanoma, breast and prostate cancer cell lines has also been described (Jones et al., 2006), and involved in the autocrine effect of tumor cell-derived RANKL on promoting cancer cell migration. Downregulation of OPG secretion has been found to be characteristic for breast cancer and multiple myeloma cells (Thomas et al., 1999; Giuliani et al., 2001). As a consequence, the RANKL-OPG balance is disturbed within the bone microenvironment in favor of supporting osteoclast-induced osteolysis and bone metastasis formation (Grimaud et al., 2003). Denosumab, a monoclonal antibody raised against RANKL, demonstrated efficacy in preventing tumor-induced bone loss in patients with skeletal metastasis (Choi et al., 2009).

It has also been reported that certain tumor cell-derived soluble factors are able to induce osteoclastogenesis independent of RANKL. Secretion of lysyl oxidase (LOX) from primary breast carcinoma cells induced osteoclast differentiation and osteolytic skeletal lesion formation in animal tumor models (Cox et al., 2015). However, LOX failed to substitute for RANKL when Tnfrsf11a (RANK)-deficient bone marrow cells were treated with recombinant LOX protein in subsequent experiments (Tsukasaki et al., 2017). Similarly, initial data indicated that Tnfrsf11a^–/–^ primary bone marrow cells were capable to differentiate into osteoclasts under TNFα stimulation, when RBP-J transcription factor was deleted in the progenitors (Kim et al., 2005; Zhao et al., 2012). However, RBP-J deletion in mice did not exhibit obvious defects in bone phenotype, suggesting that RBP-J plays a minor role in osteoclast development under physiological conditions (Zhao et al., 2012). Although there have been other factors indicated to induce RANKL-independent osteoclastogenesis, they may be able to promote osteoclast differentiation only under certain conditions and cannot completely substitute for RANK ligand (Tanaka, 2017). Identifying the precise role of these factors in osteoclasts within the solid tumor microenvironment requires further investigation.

## Role of CSF-1 Signaling in Osteoclasts and Bone Metastasis Formation

Another key osteoclastogenic cytokine that governs osteoclastogenesis is CSF-1 (or macrophage-colony stimulating factor, M-CSF). CSF-1 is a polypeptide growth factor that binds to its plasmamembrane receptor (CSF-1R) encoded by the c-fms gene (Stanley et al., 1983). CSF-1 is essential for the development and survival of preosteoclasts (Ross and Teitelbaum, 2005). CSF-1 was originally identified as a regulator of macrophages and their bone marrow precursors (Stanley et al., 1983). As shown on Figure 1, once CSF-1 binds to its transmembrane receptor CSF-1R, it induces tyrosine phosphorylation of the cytoplasmic domain of the receptor (Pixley and Stanley, 2004). Then Src homology 2 domain (SH2)-bearing adapter molecules (such as Grb2) are recruited to the phosphorylated tyrosine residues and initiate different signaling cascades (including the MAP-kinase cascade) that lead to cell proliferation and differentiation (Pixley and Stanley, 2004). CSF-1 is also known to induce cytoskeletal rearrangement in osteoclasts by activation of the c-Src and phosphoinositide 3-kinases (Nakamura et al., 1995; Insogna et al., 1997; Pilkington et al., 1998). CSF-1 deficient mice are osteopetrotic (Wiktor-Jedrzejczak et al., 1990).

When considering CSF-1R and RANK-driven non-pathological osteoclastogenesis, it is important to note, that apart from TGF-β, the cytokine IL-1 is supposed to be released from the bone as an autocrine factor. It has been described, that osteoclast precursor interaction with bone matrix (but not plastic surface) can induce osteoclast formation directly by an interleukin-1-mediated autocrine mechanism in the presence of CSF-1 (Yao et al., 2008; De Vries et al., 2015).

In addition to its physiological role in myeloid cells, increased expression of CSF-1 has been detected in breast, colorectal, ovarian and uterine cancers, where the extent of its expression correlates with poor prognosis (Kacinski, 1995; Smith et al., 1995). Furthermore, in human breast carcinomas, overexpression of CSF-1 and its receptor positively correlates with high grade invasiveness (Kacinski, 1995; Smith et al., 1995). Importantly, a strong correlation of CSF-1 expression with CSF-1R-positive tumor-associated macrophage infiltration has also been detected in human carcinomas (Tang et al., 1990; Scholl et al., 1994). We recently demonstrated that pharmacological inhibition or genetic ablation of CSF1 in cancer cells reduces the accumulation of immunosuppressive CSF-1R^+^ tumor-associated macrophages and increases CD8^+^ T cell attack on tumors (Győri et al., 2018).

In line with this, it is highly likely that certain tumor cells capable of forming osteolytic bone metastasis recruit not only tumor-associated macrophages, but monocytes/osteoclast precursors via the secretion of CSF-1 into the tumor microenvironment. Furthermore, tumor cell-derived CSF-1 can also enhance the maturation of those precursors to bone-resorbing osteoclasts in the presence of an osteoclastogenic milieu. As a consequence, pharmacological blockade of CSF-1 might offer benefits for patients with osteolytic bone metastases and CSF-1 inhibitors are being evaluated in clinical trials. PLX3397, an orally available CSF-1R inhibitor is currently being administered in clinical trials and early data suggested good tolerability and beneficial effects (Ries et al., 2015). It has also been described, that CSF-1R and αvβ3 integrins collaborate during osteoclast differentiation via shared activation of downstream signaling pathways (Faccio et al., 2003; Ross and Teitelbaum, 2005).

## Role of Integrin Signaling in Osteoclasts and Bone Metastasis Formation

Integrins are heterodimeric transmembrane proteins that facilitate cell-cell and cell-matrix interactions (Schwartz et al., 1995). Integrins can activate many intracellular signaling pathways and induce the proliferation, survival, and cytoskeletal rearrangements of the target cells (Hynes, 1992). So far 8 beta and 18 alpha integrin subunits have been described, which can combine into 24 unique heterodimers within the different cell types, each of those characterized by different ligand binding characteristics, signaling and regulatory mechanisms (Hynes, 2002). Integrin heterodimers can be activated by conformational changes in their extracellular domains. When inactive, integrin dimers are found in a closed conformation within the cell membrane. Upon activation through the cytoplasmic domains, the α and β cytoplasmic and transmembrane regions become separated resulting in the unfolding of the extracellular ligand binding domain (inside-out signaling) (Shattil et al., 2010). This open conformation then promotes extracellular ligand binding and initiates integrin-driven intracellular signaling pathways (outside-in signaling) (Qin et al., 2004).

Integrins play critical roles on both tumor cells and osteoclasts in promoting bone metastasis formation. During the process of bone resorption, mature osteoclasts attach to the bone surface, generate an actin ring mediated sealing zone and then secrete hydrochloric acid and enzymes to lyse the underlying bone matrix. All of these steps at least in part are regulated by integrin heterodimers located on the surface of osteoclasts (Teitelbaum and Ross, 2003). Different integrins are involved in the binding of osteoclasts to the bone, including αvβ3, αvβ5, and α2β1, of which, αvβ3 is the dominant integrin on osteoclasts (Ross and Teitelbaum, 2005; Novack and Teitelbaum, 2008; Ross et al., 1993). αvβ3 integrins are responsible for mediating osteoclast-bone matrix recognition and subsequent attachment to the bone surface (Ross et al., 1993). αvβ3 integrin signaling is essential for osteoclast spreading and to create the characteristic ruffled border of the plasmamembrane for subsequent resorption (Faccio et al., 2003; McHugh, 2000). Furthermore, osteopontin binding to αvβ3 induce podosome formation and cytoskeletal rearrangement (Miyauchi et al., 1991). Additional studies identified further downstream signaling effector molecules such as Vav, a critical regulator of osteoclast differentiation and actin ring formation (Faccio et al., 2005). As a consequence, mice with genetic inactivation of β3 integrin (β3^–/–^) have defective osteoclast function (McHugh, 2000) and are protected from cancer cell-induced bone loss (Bakewell et al., 2003). αvβ3 integrins are also critical for the activation of c-Src which is a key signaling molecule in osteoclast spreading and cytoskeletal reorganization (Zhang et al., 2000; Faccio et al., 2003).

Cancer cells display altered integrin expression and signaling, allowing them to colonize new tissues by escaping from cell–cell and cell–matrix connections. Maintaining adhesion to the extracellular matrix via integrins is key to cell survival (Frisch and Screaton, 2001). Disruption of cell–cell or cell–matrix interactions lead to loss of survival signals and non-transformed cells which are anchorage-dependent undergo a form of programmed cell death called anoikis (Frisch and Screaton, 2001). Under physiological conditions, this form of apoptosis assures that isolated cells are not able to migrate to inappropriate locations (Frisch and Screaton, 2001). Metastatic cancer cells that can resist anoikis utilize several different mechanisms in order to be able to settle in a novel microenvironment. These mechanisms include altered integrin expression (Bissell and Radisky, 2001), activation of integrin signaling cascade downstream molecules such as focal adhesion (Frisch et al., 1996) and c-Src kinases (Shain et al., 2002), EGFR (Demers et al., 2009), as well as suppression of apoptotic pathways (Simpson et al., 2008). Expression of αvβ3 integrin is elevated on human breast cancer bone metastases (Zhao et al., 2007), and ectopic overexpression of the β3 subunit on breast cancer cells has been demonstrated to enhance tumor establishment in bone (Sloan et al., 2006).

## Role of c-Src Kinase in Osteoclasts and Bone Metastasis Formation

The non-receptor tyrosine kinase, c-Src is a key signaling molecule in bone metabolism and plays an important role in the regulation of growth, survival, proliferation, adhesion and motility (Brunton and Frame, 2008). Preclinical studies demonstrated an important role for Src-family kinases in osteoclast-mediated bone resorption (Horne et al., 1992). Mature osteoclasts express high levels of c-Src (Miyazaki et al., 2004). Pharmacological inhibition of c-Src kinase-activity decreased preosteoclast migration and inhibited the subsequent formation of resorption pits in *in vitro* studies (de Vries et al., 2009). Mice with genetic inactivation of the c-Src gene exhibit osteopetrosis, a severe disease that makes bones abnormally dense and prone to fractures (Soriano et al., 1991). Since c-Src-deficient mice had normal osteoclast numbers, the osteopetrotic phenotype is rather due to a failure in osteoclast function (Soriano et al., 1991). Further, Src-deficient mature osteoclasts fail to form actin rings and sealing zones (Boyce et al., 1992).

Besides regulating cytoskeletal rearrangement and ruffled border formation, Src-family kinases are also present at vesicular membranes, where they are required for the secretion of hydrochloric acid and bone-degrading enzymes (Furuyama and Fujisawa, 2000; Edwards et al., 2006). Among Src-family kinase binding partners, Tks5 has been shown to mediate podosome formation and cell-cell fusion in osteoclasts (Oikawa et al., 2012). Tks5, which has been originally described as a regulator of invadopodia formation in tumor cells, was reported to be phosphorylated on tyrosine residues in a c-Src-dependent manner within osteoclasts (Oikawa et al., 2012). Furthermore, it was also shown that co-culturing malignant melanoma cells with osteoclasts promoted the formation of melanoma-osteoclast hybrid cells (Oikawa et al., 2012). Fusion of osteoclasts with cancer cells can contribute to increased bone resorption activity, secretion of chemokines promoting osteolytic bone metastasis formation and even the evasion of immune surveillance (Oikawa et al., 2012).

An elevated level of activity of c-Src is suggested to be linked to cancer progression and a large body of evidence suggests that Src-family kinase has a critical role in cancer growth and invasion (Thomas and Brugge, 1997). Further, Src expression positively correlates with the metastatic spread of cancer cells (Boyer and Poupon, 2002). Importantly, correlation was also observed between tumor cell colonization in bone and Src kinase activity (Myoui et al., 2003). Increased Src family kinase activity fueled tumor cell growth and enhanced parathyroid hormone related peptide (PTHrP) release within bone metastases (Myoui et al., 2003). Small-molecule kinase inhibitors are emerging new therapeutics in diseases with pathological bone loss and have potential for the treatment of bone metastases as well. Preclinical studies with therapeutic Src inhibitors (dasatinib, saracatinib, and bosutinib) demonstrated anti-tumor and anti-osteoclast effects, as well as clinical studies provided evidence that Src-family kinase inhibitors might be beneficial for patients with refractory disease (Boyce and Xing, 2011).

## Role of Immunoreceptor-Like Signaling in Osteoclasts and Bone Metastasis Formation

Classical immunoreceptors, such as T and B cell receptors (TCR, BCR, respectively) as well as Fc-receptors (FcR) use a common signaling machinery within the innate and adaptive immune systems. Firstly, when ligand binds to the classical immunoreceptor, tyrosine residues within the immunoreceptor tyrosine-based activation motifs (ITAMs) are phosphorylated by Src kinases as shown on Figure 1. This then results in the SH2-domain dependent recruitment of the spleen tyrosine kinase (Syk) (Fodor et al., 2006). Src and Syk non-receptor tyrosine kinases then activate downstream effector molecules such as the phospholipase Cγ2 (PLCγ2) and phosphoinositide 3-kinase (PI3K) isoforms. We and others recently recognized that this classical immunoreceptor-like signaling mechanism is present in a range of non-lymphoid cell types (e.g., osteoclasts) too (Koga et al., 2004; Mócsai et al., 2004).

Osteoclasts carry at least two ITAM sequence-containing adapter molecules, namely the DAP12 and the FcR γ-chain (FcRγ). These proteins likely work together with adhesion receptors OSCAR and TREM2 on osteoclasts (Koga et al., 2004). Deletion of DAP12 and TREM2 in mice results in failure of osteoclast differentiation and function (Paloneva et al., 2003; Humphrey et al., 2004). On the other hand, TREM2 and DAP12 deficient mice are not osteopetrotic, which indicates that osteoclastogenesis can proceed through a mechanism that requires the other ITAM-bearing molecule FcR γ-chain. FcRγ, may be able to compensate for the lack of DAP12, since double mutant mice for FcRγ and DAP12 are severely osteopetrotic (Koga et al., 2004; Mócsai et al., 2004). These ITAM-bearing co-receptors most likely mediate osteoclast-osteoblast and osteoclast-bone matrix interactions together with integrin adhesion receptors (Koga et al., 2004; Mócsai et al., 2004). More recently, paired immunoglobulin-like receptor-A (PIR-A) and osteoclast-associated receptor (OSCAR) has been shown to associate with the FcRγ-chain (FcRγ), while DAP12 interaction with signal regulatory protein β1 (SIRPβ1), sialic acid-binding immunoglobulin-like lectin 15 (Siglec-15) and myeloid DAP12-associating lectin (MDL)-1 besides TREM2 has also been reported (Koga et al., 2004; Mócsai et al., 2004; Joyce-Shaikh et al., 2010; Kameda et al., 2013; Negishi-Koga et al., 2015).

As discussed before, β3 integrin ligation leads to activation of Src-family kinases and a non-receptor kinase, Syk. The Syk tyrosine kinase is required for the differentiation and function of osteoclasts as well (Mócsai et al., 2004). Activation of Syk requires the presence of ITAM-bearing adapters, DAP12 and FcRγ-chain in the osteoclast (Mócsai et al., 2004). We recently showed, that osteoclast-specific genetic inactivation of Syk also leads to elevated bone volume in mice (Csete et al., 2019). Syk inhibitors (like fostamatinib) has shown promising effects in rheumatoid arthritis and may also provide significant bone protection in a tumor-associated environment (Mócsai et al., 2010). The osteopetrotic phenotype (Soriano et al., 1991) and defective osteoclastogenesis in the Src-deficient mice indicates that Src-family kinases might be also required for the phosphorylation of DAP12 and FcRγ in osteoclasts (Lowe et al., 1993). Syk then promotes the formation of the Bruton’s tyrosine kinase (Btk)/B cell linker protein (BLNK)/SH2 domain-containing leukocyte protein of 76 kDa (SLP76) complex, which activates the phospholipase Cγ2 (PLCγ2) enzyme. PLCγ2 cleaves the plasmamembrane phosphatidylinositol 4,5-bisphosphate (PIP_2_) into inositol 1,4,5-triphosphate (IP_3_) and diacylglycerol (DAG) (Feng et al., 2012). We and others showed that PLCγ2 is needed for the differentiation and function of osteoclasts and PLCγ2^–/–^ mice have increased bone mass (Mao et al., 2006; Chen et al., 2008; Epple et al., 2008; Kertész et al., 2012). IP_3_ generated by PLCγ2 then binds to its receptor, which in turn induces the release of calcium ions from the sarco/endoplasmic reticulum (SR/ER). Sarco/endoplasmic reticulum calcium ATPase 2 (SERCA2) re-uptakes the calcium into SR/ER (Yang et al., 2009). Repetitive influx and efflux of calcium ions results in calcium oscillations within the cytoplasm of the osteoclast. Calcium oscillations activate calcineurin, which leads to the dephosphorylation of the master regulator of osteoclastogenesis, NFATc1, enabling its autoamplification and entry to the nucleus. Calcium signaling can also induce c-Fos transcription factor via Calcium/calmodulin dependent kinase (CAMK) IV and cAMP response-element binding protein (CREB) pathways (Sato et al., 2006a). Further, transmembrane protein 64 (Tmem64) has been reported to be essential for the function of SERCA2 (Kim et al., 2013), while the transmembrane protein 178 (Tmem178) was shown to be required to suppress the excessive efflux of calcium in a PLCγ2-dependent manner (Decker et al., 2015). Accordingly, mice deficient in DAP12/FcRγ, SERCA2 and Tmem64 are osteopetrotic and exhibit defective calcium oscillations (Yang et al., 2009; Kim et al., 2013). On the other hand, lack of Tmem178 in mice results in an osteopenic phenotype with increased amplitude of calcium oscillations (Decker et al., 2015).

Immunoreceptor-like signaling has been implicated in metastatic spread and homing of cancer cells to distant organs such as the bone. TREM2 was found to be upregulated on peripheral blood monocytes in tumor-bearing hosts and elevated expression levels in pulmonary macrophages correlated with high pathological stage of lung adenocarcinomas (Yao et al., 2016). Further, DAP12 expression in colorectal cancer cells was associated with higher tumor grade (Shabo et al., 2013) and Syk is also frequently upregulated in recurrent ovarian carcinomas metastasizing to the bone (Yu et al., 2019). On the other hand, while PLCγ2 has been implicated in the proliferation and migration of several human cancers (Feng et al., 2012), its genetic deletion in mice resulted in increased tumor growth (Zhang et al., 2011). Interestingly, it has been shown that PLCγ2^–/–^ mice exhibit increased tumor mass in the bone, despite the decreased osteoclast numbers. Although PLCγ2 is not needed for T cell receptor signaling, altered CD8^+^ T cell activation was observed in PLCγ2^–/–^ tumor-bearing mice (Zhang et al., 2011). Recent evidence indicated that downregulation of PLCγ2 signaling results in the accumulation of immunosuppressive MDSCs in tumor-bearing mice instead (Capietto et al., 2013). This study highlights the role of a permissive immune phenotype and microenvironment as the requirement for osteoclast-mediated promotion of tumor growth and bone metastasis.

## Role of Ephrins and Semaphorins in Osteoclasts and Bone Metastasis Formation

Cell–cell interactions are critical for normal bone homeostasis. Ephrins and Semaphorins are known to be expressed in several cell types and regulate intercellular communication. Eph tyrosine kinase receptors and Ephrin ligands mediate interactions between bone cells through a bidirectional signaling. These membrane-bound proteins have been demonstrated to play important roles in osteoblasts and osteoclasts (Edwards and Mundy, 2008). EphrinB2 has been shown to be regulated by PTH and PTHrP in osteoblasts, while the role of EphrinA4 in modulating osteoclast activity via β3-integrin signaling has recently been identified (Stiffel et al., 2014). The altered expression of Eph tyrosine kinase receptors and ephrins has also been demonstrated in several human cancers, e.g., EphrinA2 levels were detected to be elevated in late-stage and bone metastatic prostate cancers (Lin et al., 2012).

Semaphorins play critical roles in the immune system, organ and tumor development. It has been shown that Semaphorin 3A (Sema3A) and its receptor neuropilin-1 (Nrp1) suppress osteoclast differentiation and protect mice from excessive bone loss (Hayashi et al., 2012). The Semaphorin 6D (Sema6D), which associate with TREM2/DAP12 via its receptor plexin-A1 (PlxnA1), on the other hand, promoted osteoclast differentiation and function (Takegahara et al., 2006). Semaphorins were also shown to contribute to the pathogenesis of cancer, including the regulation of epithelial-mesenchymal transitions and stem cell properties (Lontos et al., 2018). In the process of bone metastasis formation Semaphorin 4D, a coupling factor expressed on osteoclasts that inhibits osteoblast differentiation, has recently been implicated (Lontos et al., 2018).

## Role of Phosphoinositide 3-Kinases in Osteoclasts and Bone Metastasis Formation

Phosphoinositide 3-kinases (PI3Ks) generate 3-phosphoinositide lipids in cell membranes and play important roles in key biological functions including cellular proliferation, survival, cytoskeletal reorganization, migration, metabolism and vesicular trafficking (Vanhaesebroeck et al., 2012; Hawkins and Stephens, 2015). PI3Ks are subgrouped into three unique classes of which the Class I. PI3K family comprise of PI3Kα, PI3Kβ, PI3Kγ, and PI3Kδ (Hawkins et al., 2006; Vanhaesebroeck et al., 2010). PI3Kα and PI3Kβ are ubiquitously expressed, while the PI3Kγ and PI3Kδ isoforms are mainly restricted to hematopoietic lineages, e.g., white blood cells (Okkenhaug, 2013). 3-phosphoinositide lipids such as PIP_3_ can initiate a number of downstream signaling pathways and activate effector molecules, like the 3-phosphoinositide dependent protein kinase-1 (PDK1), Ser/Thr kinase Akt (or protein kinase B) and the mammalian target of rapamycin complex 1 (mTORC1) (Hawkins et al., 2006).

PI3-kinases are central downstream effectors of the RANK, CSF-1 and αvβ3 integrin receptors in osteoclasts. Activation of Class I. PI3Ks downstream of those receptors results in the generation of PIP_3_ from PIP_2_. Class I.A PI3Ks (PI3Kα, PI3Kβ, PI3Kδ) are designated based on catalytic subunits p110α, p110β, and p110δ, which associate with regulatory subunits p85α, p85β, and p55γ. The sole Class I.B member PI3Kγ consists of catalytic subunit p110γ and regulatory subunit p101 or p84. Mice lacking p110α exhibit embryonic lethality (Bi et al., 1999), while p110γ mutant mice are viable but show defects in immune cell proliferation and function (Sasaki et al., 2000). Mice knockout for the p85α/β subunit showed impaired osteoclast differentiation and ruffled border formation (Munugalavadla et al., 2008; Shinohara et al., 2012). These data indicated that PIP_3_ is a strong inducer of osteoclast differentiation and PI3-kinases might play an important role in regulating osteoclastogenesis.

We and others have shown that Class I. PI3Ks play a critical role in osteoclasts and could be potential therapeutic targets in bone metastases (Shinohara et al., 2012; Shugg et al., 2013; Győri et al., 2014). Previous studies using non-selective PI3K inhibitors (such as wortmannin) indicated a role for PI3-kinases in osteoclastogenesis (Hall et al., 1995; Nakamura et al., 1995; Sato et al., 1996). Using pharmacological and genetic approaches we demonstrated that PI3Kβ is important for normal osteoclast differentiation and function (Győri et al., 2014). PI3Kβ^–/–^ mice had increased bone mass and PI3Kβ^–/–^ osteoclasts displayed altered morphology *in vivo* (Győri et al., 2014). Further, PI3Kβ^–/–^ osteoclasts were unable to form actin rings and to release intracellular vesicles and cathepsin K (Győri et al., 2014). In addition to this, the PI3Kδ isoform, which is mainly expressed in hematopoietic cells, has been found to be an attractive target for anti-resorptive agents since recent data indicated that PI3Kδ regulates osteoclast cytoskeleton and resorptive activity (Shugg et al., 2013).

Class I. PI3Ks are often mutated in human cancers (Fruman and Rommel, 2014). Aberrant activation and amplification of PI3Kα is one of the most frequently observed mutations associated with malignant transformation (Samuels et al., 2005; Thorpe et al., 2015). Oncogenic mutations have also been detected in PI3Kβ, but rarely in PI3Kγ and PI3Kδ (Hill et al., 2010; Dbouk et al., 2013). An elevated level of activity of PI3Kδ was found in non-hematopoietic cell types, including those of breast and melanocytic origin (Sawyer et al., 2003). Further, PI3K downstream targets are also often seen abnormally activated in human malignancies. Overexpression of Akt/PKB has been observed in a wide range of solid tumors, including breast, ovarian and prostate carcinomas (Scheid and Woodgett, 2001; Vivanco and Sawyers, 2002). The recognition of the tumor suppressor PTEN, a phosphatase for 3-phosphoinositide lipids, proved to be an indicator of aberrant PI3K signal transduction in cancer (Cantley and Neel, 1999). Deletions and mutations in the PTEN gene also lead to the accumulation of PI3K lipid products, which is observed in many human tumors (Di Cristofano and Pandolfi, 2000).

There is substantial interest in the pharmaceutical industry to develop PI3K inhibitors for the treatment of human cancers. To avoid the serious side effects of pan–Class (I) PI3K drugs affecting glucose homeostasis (Busaidy et al., 2012), administration of isoform-selective PI3K inhibitors below their maximum tolerated dose, most likely in combination may be beneficial. Based on its role in the tumor microenvironment PI3Kβ and/or PI3Kδ may be suitable therapeutic targets for bone metastases with tumor-induced osteolysis.

## Role of Micrornas and Extracellular Vesicles in Osteoclasts and Bone Metastasis Formation

MicroRNAs are non-coding RNAs of approximately 20–22 nucleotides in length which regulate critical cellular processes including proliferation, differentiation, and survival. Recent data indicated that microRNAs are involved in tumorigenesis and osteolytic bone metastasis formation as well. Various tumor-cell derived mature microRNAs were implicated in regulating osteoclast differentiation. miR-223 was shown to regulate the level of the CSF1 receptor in preosteoclasts (Sugatani and Hruska, 2007), while miR-503 targets RANK (Chen et al., 2014). Further, miR-29 promotes murine osteoclastogenesis by regulating osteoclast commitment and migration (Franceschetti et al., 2013). On the other hand, miR-124 was detected to suppress transcription factor NFATc1 during osteoclastogenesis (Lee et al., 2013), while miR-155 represses MITF and PU.1 transcription factors (Mann et al., 2010). It has been reported that administration of miR-141 and miR-219 results in a significant drop in the number of bone-resorbing osteoclasts *in vivo* and tumor burden in breast carcinoma-bearing mice (Ell et al., 2013). Similarly, miR-33a suppressed bone metastasis formation by targeting PTHrP and altering the RANKL/OPG ratios in osteoblasts (Kuo et al., 2013).

MicroRNAs were recently revealed to be present in extracellular vesicles (Valadi et al., 2007). Extracellular vesicles are lipid bilayer coated vesicles ranging from approximately 50 to 5000 nm in diameter. Three main types of extracellular vesicles exist: microvesicles, exosomes and apoptotic bodies (Marton et al., 2017). MicroRNA-containing extracellular vesicle secretion depends on various different factors. Numerous studies suggested the involvement of extracellular vesicles secreted by tumor cells in the development of osteolytic bone metastases (Kagiya, 2015). Exosomal miR-21, miR-210 and miR-378 were found to be important for regulating osteoclast differentiation (Kagiya, 2015). Further, miR-16 and miR-378 were detected to be higher in the serum of metastatic breast cancer patients (Arroyo et al., 2011; Ell et al., 2013). Accordingly, extracellular vesicles play an important role in intercellular communication via the transfer of not only microRNAs and proteins, but also bioactive lipids such as PIP_3_ generated by phosphoinositide 3-kinases.

## Therapeutic Aspects and Future Perspectives

Tight coupling between bone formation and resorption is essential for bone remodeling. Disruption of this balance can lead to skeletal disorders. Skeletal metastatic disease is a severe consequence of tumor cell dissemination from primary cancer sites, and significantly decreases wellbeing and life expectancy in patients. The bone is a major target for tumor metastasis as it provides a unique environment, which promotes solid cancer cell dissemination (Weilbaecher et al., 2011). The balance between bone-forming osteoblasts and bone-resorbing osteoclasts is altered by bone metastasis formation (Mundy, 2002). Cancer cell invasion into the bone is associated with the activation of osteoclasts. Osteoclast-mediated osteolysis then leads to the release of cytokines and growth factors deposited in the bone matrix. This further fuels tumor growth and dissemination within the tissue (“vicious cycle” of bone metastasis formation) (Faccio, 2011).

**FIGURE 2:**
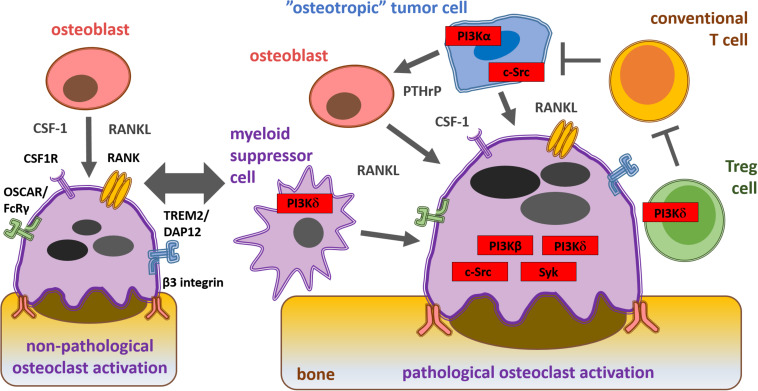
Schematic representation of physiological and pathological osteoclast activation during bone metastasis formation and key signaling molecules within the solid tumor microenvironment from a therapeutic aspect. Osteoblast-derived cytokines and interactions with the bone matrix lead to physiological osteoclast activation via CSF1, RANK, costimulatory and β3 integrin receptors. “Osteotropic” tumor cell recruited regulatory T cells (Tregs) and myeloid derived suppressor cells (MDSC) inhibit conventional T cells (Tconv) and promote cancer cell-induced pathological osteoclast activation via PI3Kβ, PI3Kδ, c-Src, and Syk.

RANK ligand, an important osteoclastogenic factor, is expressed by osteoblasts and cancer cells (Kearns et al., 2008). PTHrP secreted by tumor cells indirectly promotes osteoclastogenesis-supporting cells to express RANK ligand (Suva et al., 1987; Mancino et al., 2001). CSF-1, necessary for the development of preosteoclast to osteoclasts, is also derived from cancer cells (Stanley et al., 1983; Smith et al., 1995). Once bone metastasis is established, cancer cells recruit other tumor-associated cells, including fibroblasts and immune cells which secrete cytokines (such as IL-17, IL-1, IL-6, and TNF-α) that increase RANK ligand expression (Mancino et al., 2001; Kitazawa and Kitazawa, 2002). Integrin and immunoreceptor-like signaling leads to the activation of key effector molecules, such as Src-family kinases (Myoui et al., 2003), Syk non-receptor tyrosine kinase (Mócsai et al., 2010) and phosphoinositide 3-kinases (PI3Ks), which further enhance osteoclast activity and subsequent bone destruction (Di Cristofano and Pandolfi, 2000). Summary of the mechanisms of osteoclast activation during cancer cell-induced osteolysis as well as the key osteoclast signaling molecules are shown on Figure 2.

Better understanding of the molecular mechanisms governing osteoclast activation in tumor induced-osteolysis and bone metastasis formation may result in the development of novel therapeutic approaches. As several of the osteoclast signal transduction pathways are also activated in cancer cells, they might be tackled synergistically within the solid tumor microenvironment resulting in a parallel reduction in cancer growth and osteoclast-mediated bone loss. On the other hand, a precision medicine-based approach is required to overcome cancer cell resistance mechanisms and to minimize side effects and interference with the mechanisms of physiological bone homeostasis. Taken together, pharmacological targeting of osteoclast and tumor cell signaling pathways may provide novel approaches to improve the long-term survival of cancer patients with bone metastases.

## Author Contributions

All authors listed have made a substantial, direct and intellectual contribution to the work, and approved it for publication.

## Conflict of Interest

The authors declare that the research was conducted in the absence of any commercial or financial relationships that could be construed as a potential conflict of interest.
